# Ready-To-Eat Rocket Salads as Potential Reservoir of Bacteria for the Human Microbiome

**DOI:** 10.1128/spectrum.02970-22

**Published:** 2022-12-20

**Authors:** Giacomo Mantegazza, Giorgio Gargari, Robin Duncan, Fabio Consalez, Valentina Taverniti, Patrizia Riso, Simone Guglielmetti

**Affiliations:** a Division of Food Microbiology and Bioprocesses, Department of Food, Environmental, and Nutritional Sciences, Università degli Studi di Milano, Milan, Italy; b Division of Human Nutrition, Department of Food, Environmental, and Nutritional Sciences, Università degli Studi di Milano, Milan, Italy; University of Minnesota—Twin Cities

**Keywords:** *Leuconostoc*, rocket salad, 16S rRNA gene profiling, nonhuman microbiota, raw vegetables, lactic acid bacteria, microbial depletion hypothesis

## Abstract

Reportedly, Western-type diets may induce the loss of key microbial taxa within the gastrointestinal microbiota, promoting the onset of noncommunicable diseases. It was hypothesized that the consumption of raw vegetables could contribute to the maintenance of the intestinal microbial community structure. In this context, we explored bacteria associated with commercial rocket salads produced through different farming practices: traditional (conventional, organic, and integrated) and vertical farming. Viable counts of mesophilic bacteria and lactic acid bacteria (LAB) were performed on plate count agar (PCA) and de Man-Rogosa-Sharpe (MRS) agar at pH 5.7, whereas metataxonomics through 16S rRNA gene sequencing was used to profile total bacteria associated with rocket salads. We found that rocket salads from vertical farming had much fewer viable bacteria and had a bacterial community structure markedly different from that of rocket salads from traditional farming. Furthermore, although α- and β-diversity analyses did not differentiate rocket samples according to farming techniques, several bacterial taxa distinguished organic and integrated from conventional farming salads, suggesting that farming practices could affect the taxonomic composition of rocket bacterial communities. LAB were isolated from only traditional farming samples and belonged to different species, which were variably distributed among samples and could be partly associated with farming practices. Finally, the INFOGEST protocol for *in vitro* simulation of gastrointestinal digestion revealed that several taxonomically different rocket-associated bacteria (particularly LAB) could survive gastrointestinal transit. This study suggests that commercial ready-to-eat rocket salads harbor live bacteria that possess the ability to survive gastrointestinal transit, potentially contributing to the taxonomic structure of the human gut microbiota.

**IMPORTANCE** Western-type diets are composed of foods with a reduced amount of naturally occurring microorganisms. It was hypothesized that a microbe-depleted diet can favor the alteration of the human intestinal microbial ecosystem, therefore contributing to the onset of chronic metabolic and immune diseases currently recognized as the most significant causes of death in the developed world. Here, we studied the microorganisms that are associated with commercial ready-to-eat rocket salads produced through different farming practices. We showed that rocket salad (a widely consumed vegetal food frequently eaten raw) may be a source of lactic acid bacteria and other microbes that can survive gastrointestinal transit, potentially increasing the biodiversity of the intestinal microbiota. This deduction may be valid for virtually all vegetal foods that are consumed raw.

## INTRODUCTION

Since the discovery of their existence, microorganisms have been regarded mainly as causes of food spoilage and etiological agents of diseases. Therefore, preservation and sanitation methods have been adopted, in conjunction with constantly improved hygiene and farming practices, to counteract the presence and proliferation of microbes in food. However, the same strategies adopted to contrast harmful microbes inevitably get rid of harmless microbial populations associated with food. This consequence of hygiene practices might be relevant in the context of the so-called microbial depletion hypothesis (also known as the “old-friends hypothesis,” which evolved from the original “hygiene hypothesis”), according to which reduced exposure to microorganisms from the environment and food could have contributed to increases in the incidences of both immune diseases and allergic disorders (1).

In a more general perspective, recent studies suggest that the drastic shift in lifestyle that occurred with industrialization, which is characterized by the intensive use of antibiotics, extensive sanitation, and the wide diffusion of processed foods, has impacted the human gut microbiota composition, resulting in a population-wide reconfiguration of microbial ecosystems, which contributes to the onset of chronic metabolic and immune diseases currently recognized as the most significant cause of death in the developed world (2–4). Notably, increasing experimental evidence demonstrates that the dietary constituents characterizing Western-style diets, such as simple sugars, saturated fats, sodium chloride, and synthetically produced or natural additives, have direct effects on the intestinal microbiota community structure, promoting several detrimental metabolic consequences in the host (5). For instance, the increased intake of sodium chloride was demonstrated to reduce indole-producing lactobacilli in the gut, favoring Th17 skewing and consequently resulting in increased blood pressure (6). Furthermore, the commonly used emulsifiers carboxymethyl cellulose and polysorbate 80 were shown to erode the host’s mucous layer and induce low-grade inflammation, metabolic syndrome, and colitis in mice (7). In addition, several studies reported that an enhanced intake of dietary fat and simple sugars, as occurs in Western-pattern diets, *per se* can change the composition and functionality of the microbiota, increase gut permeability, promote metabolic endotoxemia, and trigger systemic (low-grade) inflammation that promotes the development of metabolic dysregulation (8, 9).

A dominant contribution to urban-induced alterations in the gut microbiota is therefore provided by diet, and contextually, the integration of Western-style dietary patterns with fermented foods (i.e., “foods made through desired microbial growth and enzymatic conversions of food components” [10]) was suggested as a potentially effective strategy to counteract the detrimental consequences of bacterial deprivation in the environment and in food (11). Most consumed fermented foods exclusively or predominantly harbor lactic acid bacteria (LAB), which are the microorganisms most studied and recognized for their health-promoting properties (12). However, unfermented foods that are eaten raw, although they may contain fewer microbial cells than fermented foods, may also supply LAB to the human orogastrointestinal tract, in addition to many other microorganisms with a much wider taxonomic representation (13). Raw vegetables, for instance, can reach our table delivering microbes derived from both their leaf microbiota and the soil. Interestingly, *in vivo* animal studies demonstrated that soil bacteria can increase gut microbial diversity and influence immune health, specifically enhancing innate immunity (14, 15). Consequently, it can be speculated that greater diversity in microbial cells ingested with food can contribute to an increase in the biodiversity of the gut microbiota, enhancing its ability to rebalance host immune homeostasis. In addition, gut bacteria seem to contribute to the production of essential vitamins and nutrient absorption (e.g., inducing host genes for nutrient uptake, affecting the gastrointestinal transit time) (16, 17).

In the context described above, the present study aims to test the hypothesis that vegetables eaten raw can be a nonnegligible source of microorganisms for the human gastrointestinal tract. Specifically, both viable plate counts and metataxonomics were used to explore the bacterial taxonomic diversity (with a specific focus on LAB) of commercial rocket salads (a widely consumed vegetal food frequently eaten raw and most often commercially available as a ready-to-eat product) produced through different farming practices. In addition, the potential ability of rocket salad-associated bacteria to survive gastrointestinal transit was also assessed using an *in vitro* gastrointestinal digestion test.

## RESULTS

### Taxonomic profiling of rocket salad-associated bacteria.

Illumina MiSeq sequencing of 16S rRNA gene amplicons produced a total of 4,363,400 reads (mean ± standard deviation [SD], 69,260 ± 21,252 reads). After processing and denoising, a total of 1,024,011 (16,254 ± 4,942) merged reads were obtained. Next, after the depletion of sequences associated with chloroplasts and mitochondria, a mean of 2,984 cleaned reads per sample was found.

**FIG 1:**
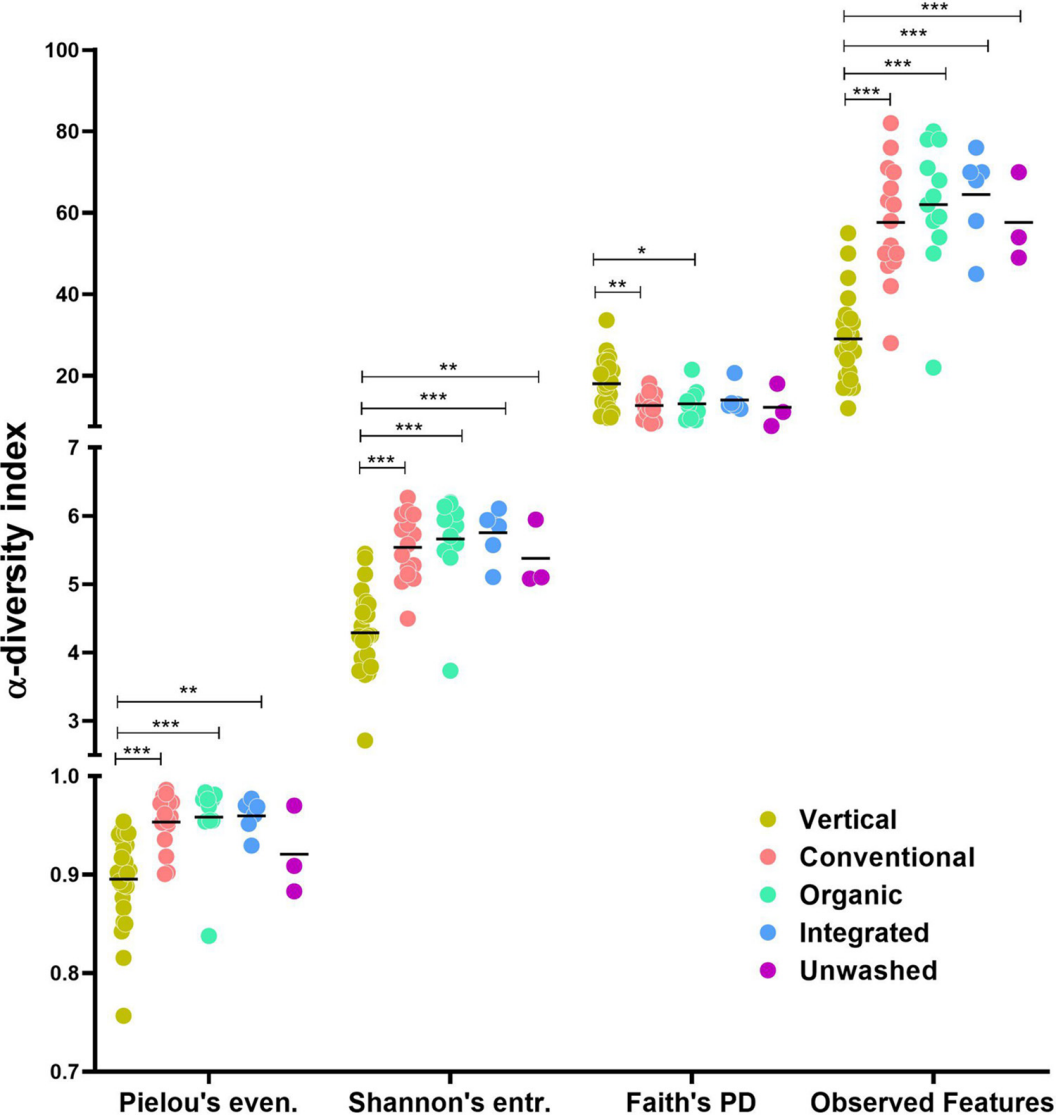
Scatter dot plot representing the intrasample (α) diversity analysis of 16S rRNA gene profiling data for rocket salad samples carried out with four different indices: Pielou’s evenness, Shannon’s entropy, Faith’s phylogenetic diversity, and observed-feature taxonomic richness. Each color refers to the farming strategy for the rocket samples.

After taxonomic assignment, 16S rRNA gene profiling data were used to assess the biodiversity of the bacterial communities associated with rocket salads. The analysis of α-diversity was carried out with four indices that differently consider the evenness, richness, and phylogenetic distance of the bacterial taxa within each sample. Pielou’s (measure of evenness), Shannon’s entropy (considering both evenness and richness), and observed-feature (measure of richness) indices were significantly lower in vertical farming than in traditional farming salads (*P* < 0.001), whereas the opposite result was found for Faith’s phylogenetic diversity (PD) index, which is a measure of biodiversity based on phylogeny (Fig. 1). No significant differences were found among traditional farming salads. Therefore, α-diversity analysis revealed that the bacteria associated with rocket salads from vertical farming had a significantly reduced taxonomic richness, were more unevenly distributed, and possessed a wider phylogenetic distance.

**FIG 2:**
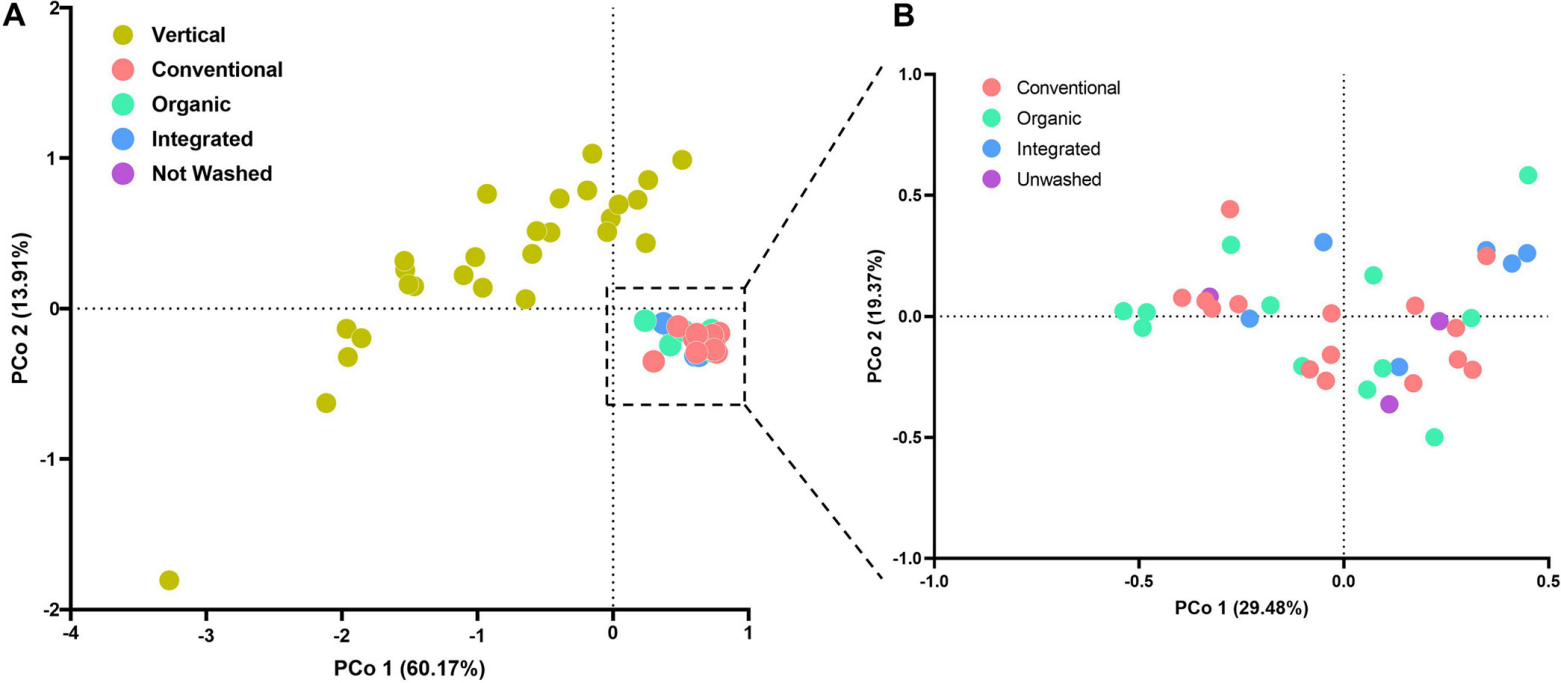
Intersample (β) diversity of the microbiota associated with rocket salads shown as data from principal-coordinate analysis of weighted UniFrac distances based on amplicon sequence variant (ASV) abundances. (A) Analysis performed with all investigated rocket salad samples. (B) Analysis performed with rocket samples from traditional farming only. The first two coordinates (PCo1 and PCo2) are displayed, with the percentages of explained variance in parentheses.

The marked difference in the bacterial community structures between vertical and traditional farming rocket salads was also evidenced by β-diversity analysis based on the weighted UniFrac algorithm (Fig. 2). A principal-coordinate analysis (PCoA) plot of weighted UniFrac distances also revealed much higher intersample diversity among vertically farmed salads than among the traditionally farmed ones (Fig. 2A). On the contrary, samples from traditional farming could not be distinguished according to weighted UniFrac distances and showed lower intersample diversity in their bacterial community structures (Fig. 2B). Similar results were found when intersample diversity was studied by unweighted and generalized (α = 0.5) UniFrac distances (not shown).

**FIG 3:**
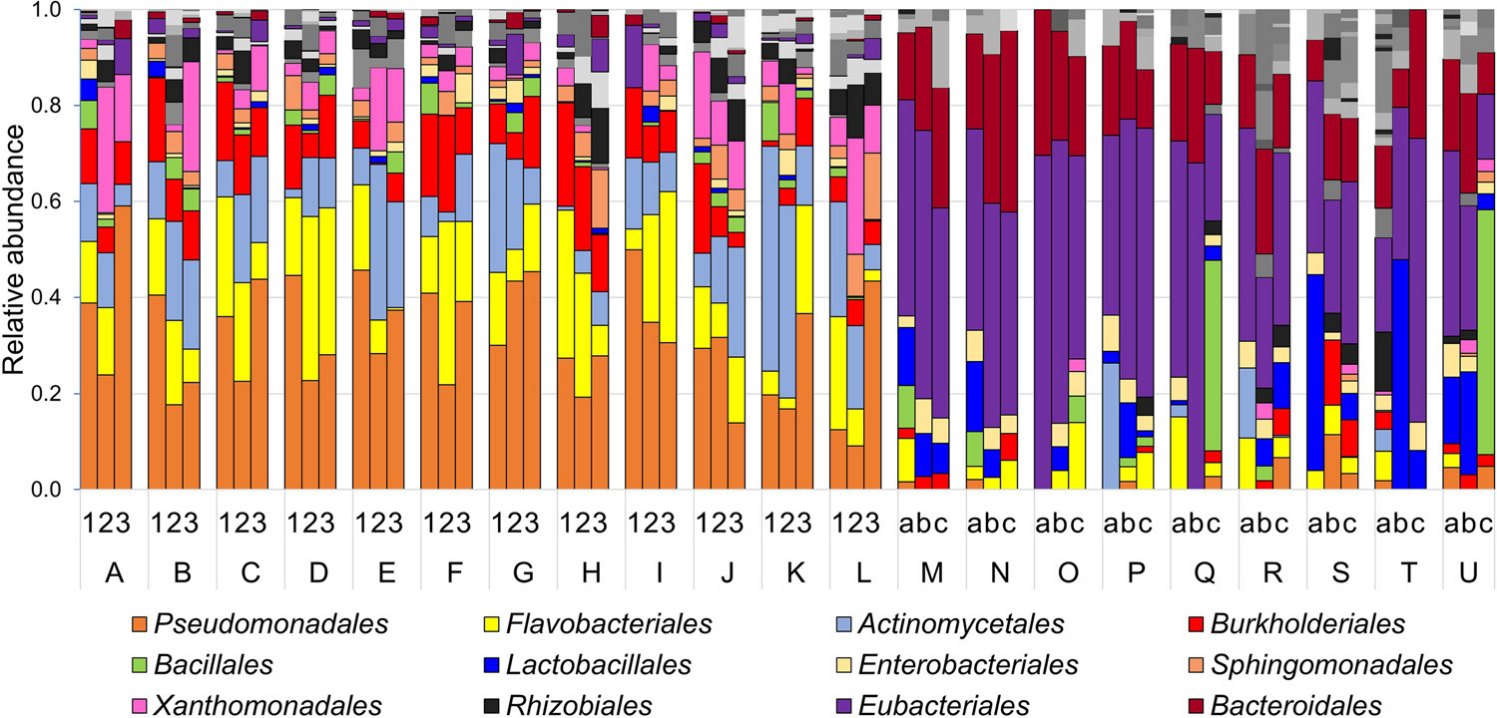
Order-level composition of the bacterial communities associated with rocket salad samples investigated in this study. Sample labels are according to the scheme represented in Fig. 9.

The analysis of bacterial taxon abundances showed that the microbiota associated with vertical farming samples was markedly different from those of other samples because it was dominated by the orders *Eubacteriales* (formerly named *Clostridiales*) (phylum *Firmicutes*), *Bacteroidales* (*Bacteroidetes*), and *Lactobacillales* (*Firmicutes*), whereas traditional farming rocket salads principally harbored the Gram-negative orders *Pseudomonadales* (class *Gammaproteobacteria*), *Burkholderiales* (class *Betaproteobacteria*), and *Flavobacteriales* (phylum *Bacteroidetes*) and the Gram-positive order *Actinomycetales* (phylum *Actinobacteria*) (Fig. 3). At the genus level, 67% of samples from vertical farming (*n* = 18) had undefined *Eubacteriales* as the most abundant genus; *Lactobacillus* and *Bacillus* were the dominant genera in the other 3 and 2 vertical farming samples, respectively; finally, the remaining 4 vertical farming samples had 1 of the following 4 genera as the most abundant genus: undefined *Microbacteriaceae*, Acinetobacter, undefined *Rikenellaceae*, and undefined *Chitinophagaceae* (see Fig. S1 in the supplemental material). On the contrary, all rocket salads from traditional farming had Pseudomonas (belonging to the phylum *Proteobacteria*) and *Flavobacterium* (*Bacteroidetes*) as the most abundant genera, with the addition of an undefined *Xanthomonadaceae* genus (*Proteobacteria*) (the most abundant in 3 samples), *Rhodococcus* (*Actinobacteria*) (the most abundant in 2 samples), and *Arthrobacter* (*Actinobacteria*) (the most abundant in 1 sample) (Fig. S1).

**FIG 4:**
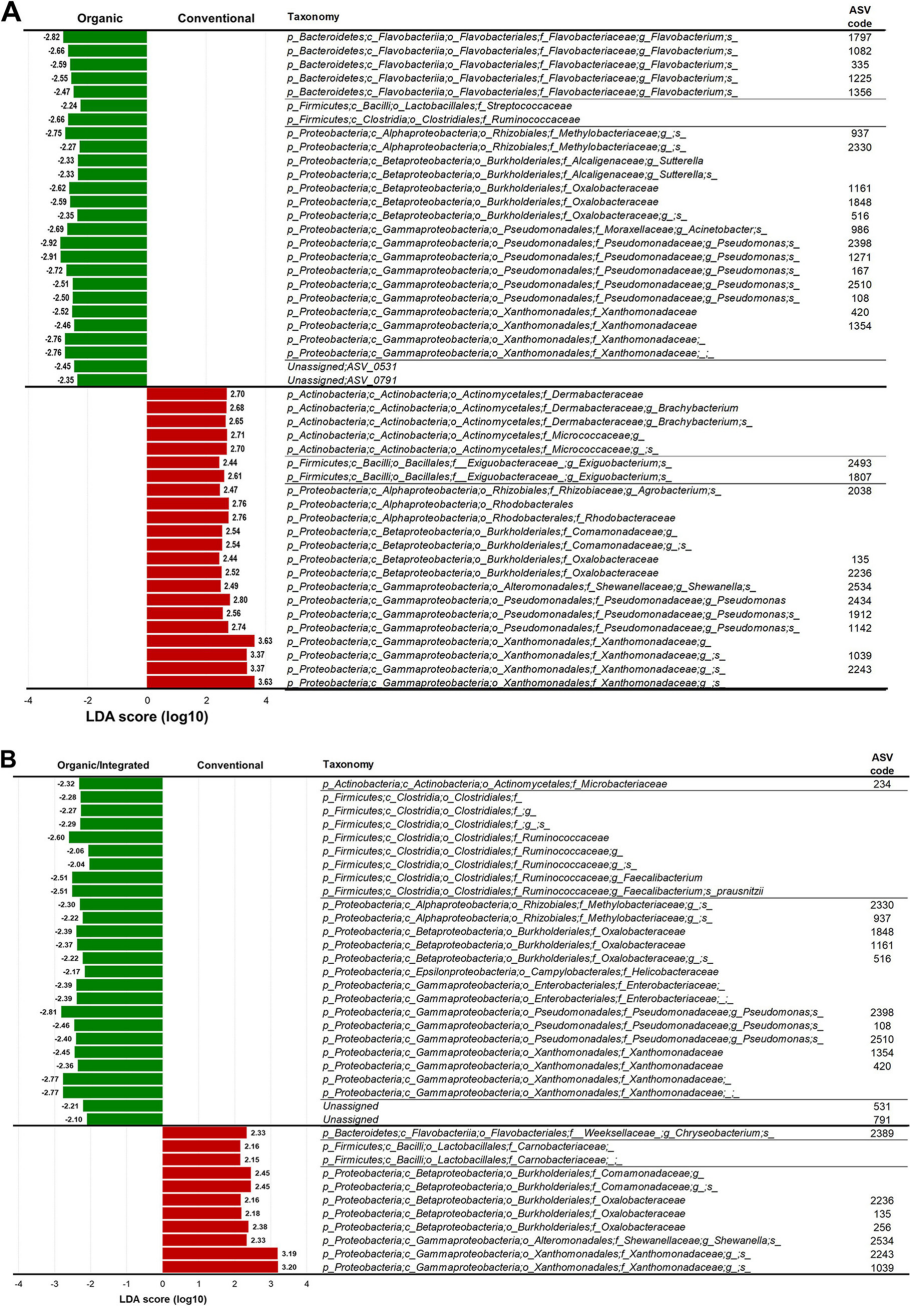
Differentially abundant bacterial taxa in rocket salads obtained with different traditional farming practices identified using the linear discriminant analysis (LDA) effect size (LEfSe) algorithm (*P* < 0.05). (A) Conventional versus organic farming samples. (B) Conventional versus organic/integrated farming samples. The taxonomic lineage of each taxon according to the Greengenes database is shown. p, phylum; c, class; o, order; f, family; g, genus; s, species; ASV, amplicon sequence variant.

Subsequently, using the linear discriminant analysis (LDA) effect size (LEfSe) algorithm, we analyzed the relative abundances of the bacterial groups found in traditional farming samples to identify potential taxonomic units that can discriminate between conventional farming (*n* = 5 commercial products at 3 time points each) and organic (Org) farming (*n* = 4 commercial products at 3 time points each) rocket salads. This analysis revealed that 26 bacterial taxa were significantly more abundant in organic rocket salads, whereas 22 taxa were overrepresented in conventional salads (Fig. 4A). More specifically, organic farming samples were enriched in taxa belonging to the *Bacteroidetes* genus *Flavobacterium* and were characterized by higher abundances of the *Firmicutes* families *Streptococcaceae* and *Ruminococcaceae* and the *Proteobacteria* genus *Sutterella*. In contrast, conventional farming salads were enriched in taxa belonging to the *Actinobacteria* families *Dermabacteraceae* and *Micrococcaceae* and had significantly higher abundances of the *Proteobacteria* family *Rhodobacteraceae*. In addition, organic and conventional farming samples were represented by different amplicon sequence variants (ASVs) ascribed to the genus Pseudomonas (Fig. 4A). The same analysis was also performed by including the integrated (Int) farming salad samples (*n* = 2 commercial products at 3 time points each) in the organic farming group (Org/Int group). The obtained results revealed that 26 bacterial taxa were overrepresented in the Org/Int salads, whereas 11 taxa were enhanced in conventional salads (Fig. 4B). In particular, in the Org/Int samples, we observed increased abundances of several bacterial groups belonging to the order *Eubacteriales* (including the family *Ruminococcaceae* and the species Faecalibacterium prausnitzii), an undefined member of the family *Enterobacteriaceae*, and the family *Helicobacteraceae*. On the contrary, conventional farming salads were enriched in an undefined member of the *Firmicutes* family *Carnobacteriaceae* and an undefined member of the *Proteobacteria* family *Comamonadaceae* (Fig. 4B).

Overall, metataxonomic analysis showed that the microbiota associated with vertical farming rocket salads had a bacterial community structure that was markedly different from that of rocket salads from traditional farming. Furthermore, although α-diversity and intersample diversity analyses did not differentiate rocket samples according to farming techniques, several specific taxonomic units were found to distinguish organic and integrated from conventional farming salad samples, suggesting that farming practices could affect the taxonomic composition of bacterial communities associated with rocket salad.

### Viable counts of rocket salad-associated bacteria.

**FIG 5:**
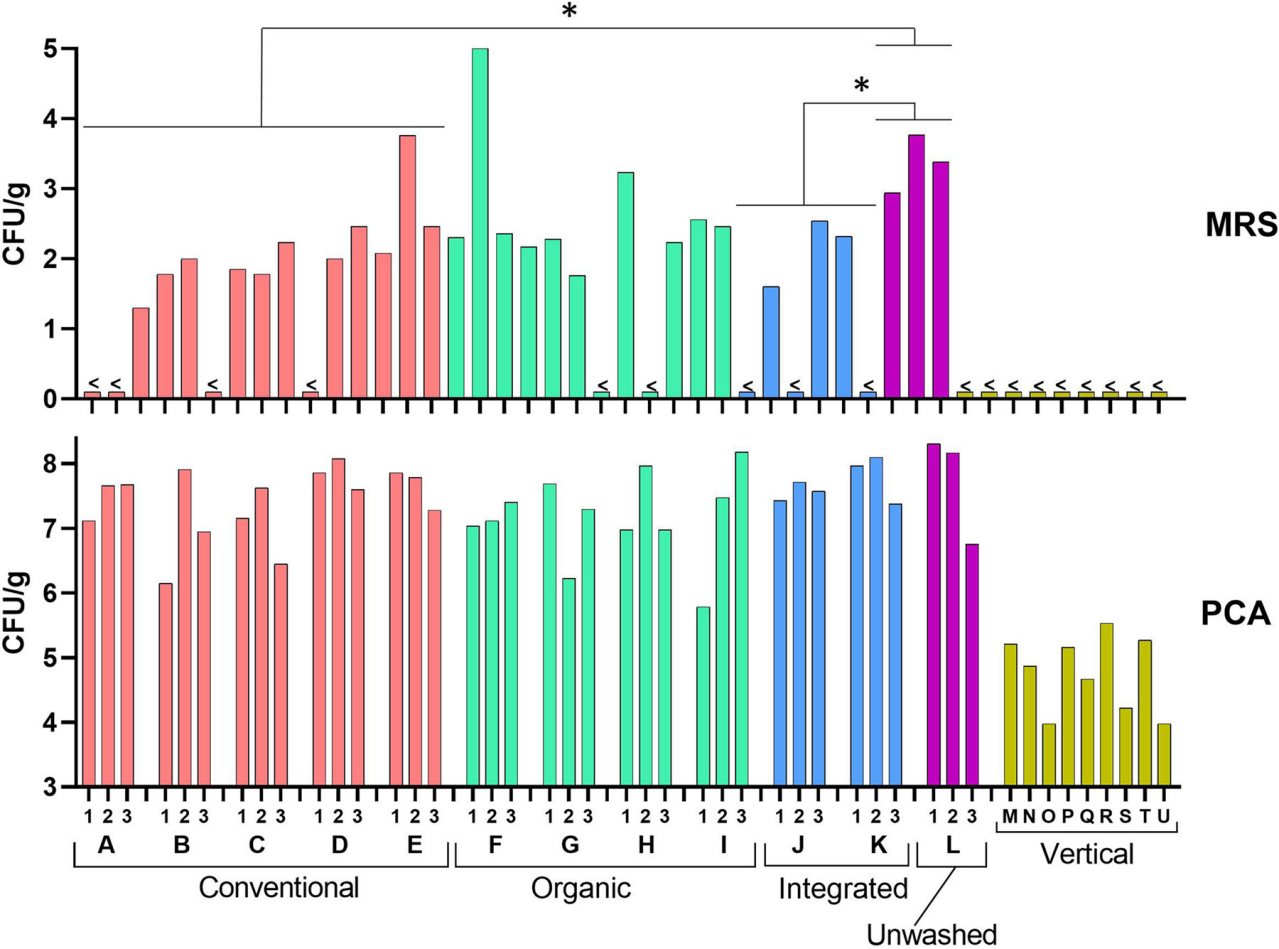
Viable cell counts of bacteria associated with rocket salads determined on plate count agar (PCA) and de Man-Rogosa-Sharpe (MRS) agar, expressed as the number of CFU per gram of salad. Sample labels are according to the scheme represented in Fig. 9. <, under the detection limit (100 CFU per g of rocket salad). Statistics are according to unpaired Student’s *t* test. *, *P* < 0.05.

Agar plate count experiments revealed that rocket salads from traditional farming harbored much more bacteria than rocket salads from vertical farming according to the number of colonies enumerated on both plate count agar (PCA) (mean ± standard deviation, 7.41 ± 0.60 versus 4.77 ± 0.59 CFU/g [*P* < 0.001]) and de Man-Rogosa-Sharpe (MRS) (1.88 ± 1.24 versus 0 CFU/g) media (*P* < 0.05) (Fig. 5). No differences were found when comparing the impacts of the different traditional farming methods on viable cell counts on PCA (conventional, 7.41 ± 0.56 CFU/g; organic, 7.18 ± 0.67 CFU/g; integrated, 7.70 ± 0.29 CFU/g; unwashed, 7.74 ± 0.86 CFU/g). Regarding the viable counts of lactic acid bacteria (LAB), more colonies were found in unwashed rocket salads (3.37 ± 0.41 CFU/g) than in rocket salads from conventional (1.61 ± 1.08 CFU/g [*P* < 0.05]) and integrated (1.08 ± 1.22 versus 3.37 ± 0.41 CFU/g [*P* < 0.05]), but not organic (2.20 ± 1.03 CFU/g [*P* = 0.16]), farming.

Overall, viable cell counts demonstrated that rocket salad from vertical farming was characterized by a much lower viable bacterial load and the absence of detectable viable LAB. In addition, unwashed rocket salad from conventional agriculture had a bacterial load not significantly dissimilar from that of ready-to-eat rocket salads from traditional farming, but it harbored significantly more LAB.

### Taxonomic characterization of lactic acid bacteria isolated from rocket salad.

**FIG 6:**
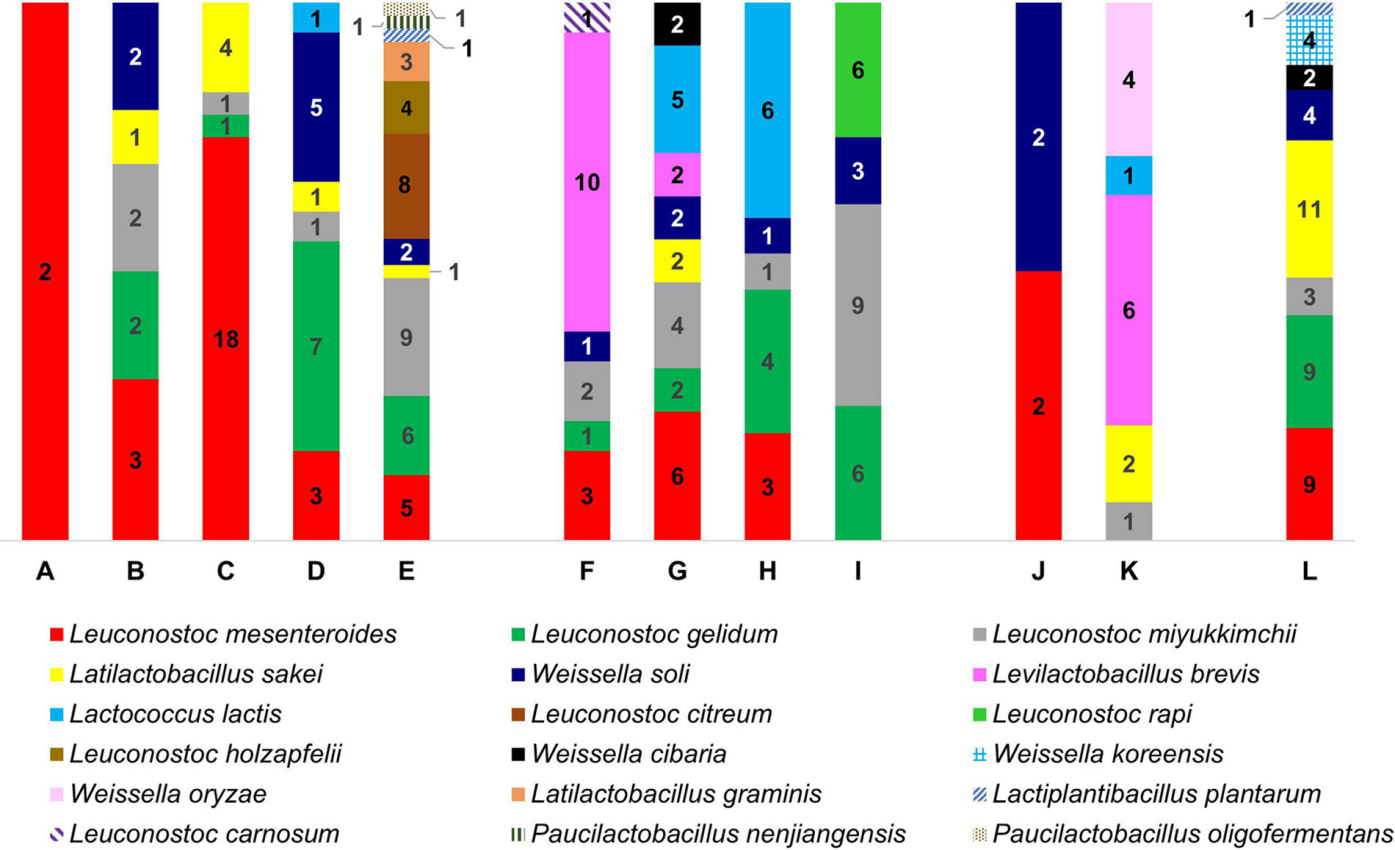
Histogram summarizing the species of lactic acid bacteria (LAB) isolated from rocket salads. The numbers in the graph indicate the isolates of each LAB species in any rocket salad product. The letters A to L refer to the traditional farming rocket salads as shown in Fig. 9 and Fig. S2 in the supplemental material.

**FIG 7:**
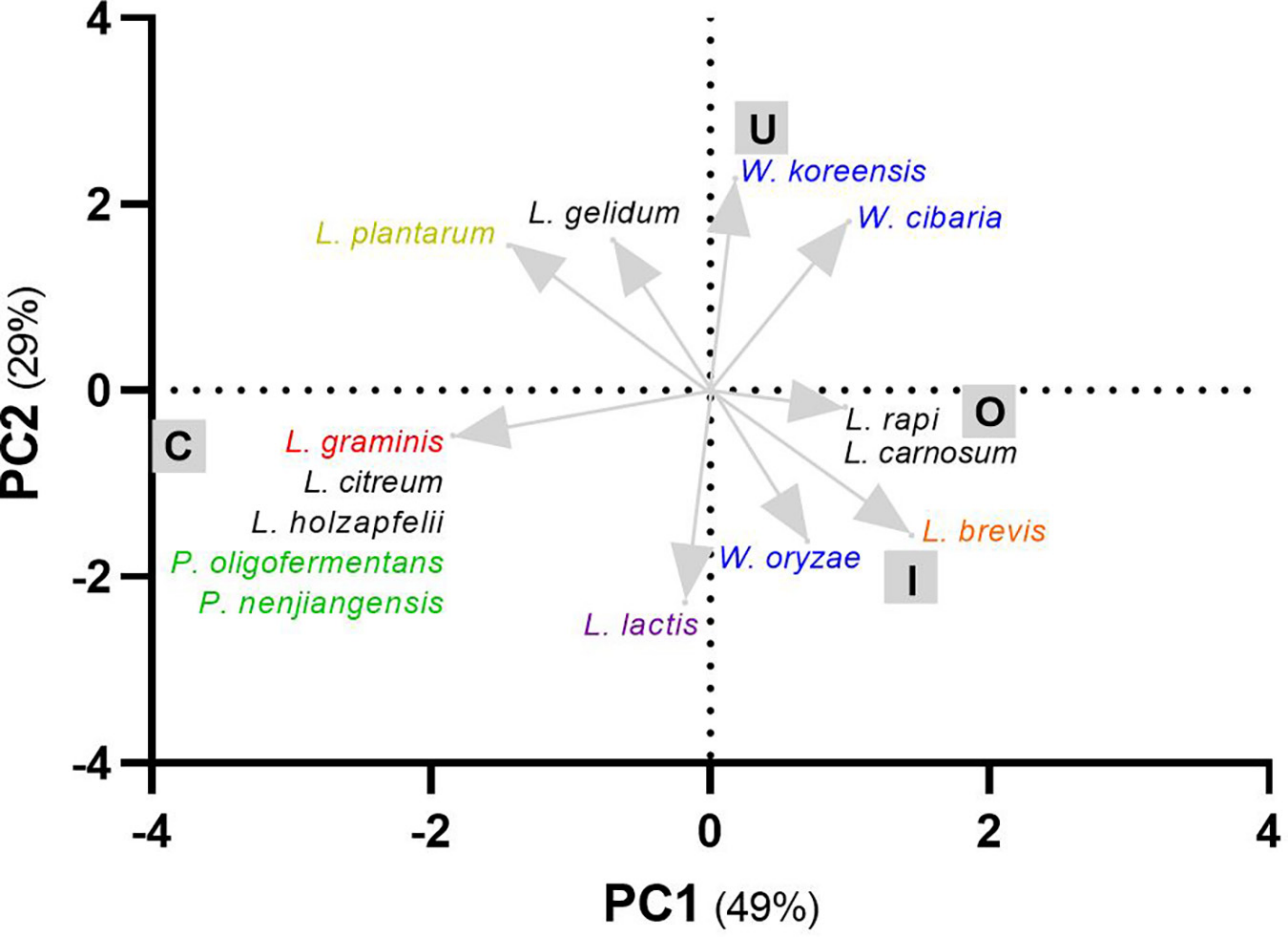
Principal-component analysis based on the presence-absence matrix of lactic acid bacterial species isolated from rocket salads as reported in Fig. 6 and Fig. S2 in the supplemental material. C, conventional; I, integrated; O, organic; U, unwashed. The color of the taxonomic name refers to the genus: black, *Leuconostoc*; blue, *Weissella*; green, *Paucilactobacillus*; red, *Latilactobacillus*; yellow, *Lactiplantibacillus*; violet, *Lactococcus*; orange, *Levilactobacillus*.

In order to define the taxonomic distribution of viable LAB associated with rocket salad, we isolated 237 colonies (95 from conventional, 84 from organic, 18 from integrated, and 43 from unwashed rocket salads) from MRS agar plates. Sequencing of the 16S rRNA gene amplified by PCR from each isolate revealed that all isolates belonged to LAB species, with the only exception being two isolates from an organic farming salad sample that were taxonomically assigned to the *Proteobacteria* species Herbaspirillum huttiense. *Leuconostoc* was the prevalent genus in conventional (76% of the isolates), organic (59%), and unwashed (49%) rocket salads. On the contrary, *Levilactobacillus* and *Weissella* were the most frequently found genera among isolates from integrated farming (33%) rocket samples (Fig. 6; Fig. S2). Less represented genera were *Latilactobacillus* (25 isolates), *Lactococcus* (16 isolates, 14 of which were from organic salads), *Lactiplantibacillus* (2 isolates), and *Paucilactobacillus* (2 isolates). In total, the isolates were ascribed to 18 different LAB species, none of which were found in all samples. Only four species were found in all kinds of salads (conventional, organic, integrated, and unwashed): Latilactobacillus sakei, Leuconostoc mesenteroides, Leuconostoc miyukkimchii, and Weissella soli (Fig. 6; Fig. S2). Principal-component analysis based on a presence-absence matrix of bacterial species revealed that conventional salads could be distinguished from the others mostly according to the presence of Latilactobacillus graminis, Leuconostoc citreum, Leuconostoc holzapfelii, Paucilactobacillus oligofermentans, and Paucilactobacillus nenjiangensis, whereas the species Leuconostoc rapi and L. carnosus described organic rocket salads. In addition, Levilactobacillus brevis and Weissella oryzae were the species that characterized integrated salads, whereas Weissella koreensis and W. cibaria were associated with unwashed salads (Fig. 7).

Overall, these results indicate that approximately all colonies isolated on MRS agar (96% of isolates) are ascribable to LAB taxa and predominantly belong to the genus *Leuconostoc* (60% of isolates). In addition, the LAB associated with rocket salads from traditional farming belong to several different LAB species, which are variably distributed among salad samples and can be partly associated with farming practices.

### Survival of rocket-associated bacteria during simulated gastrointestinal transit.

**FIG 8:**
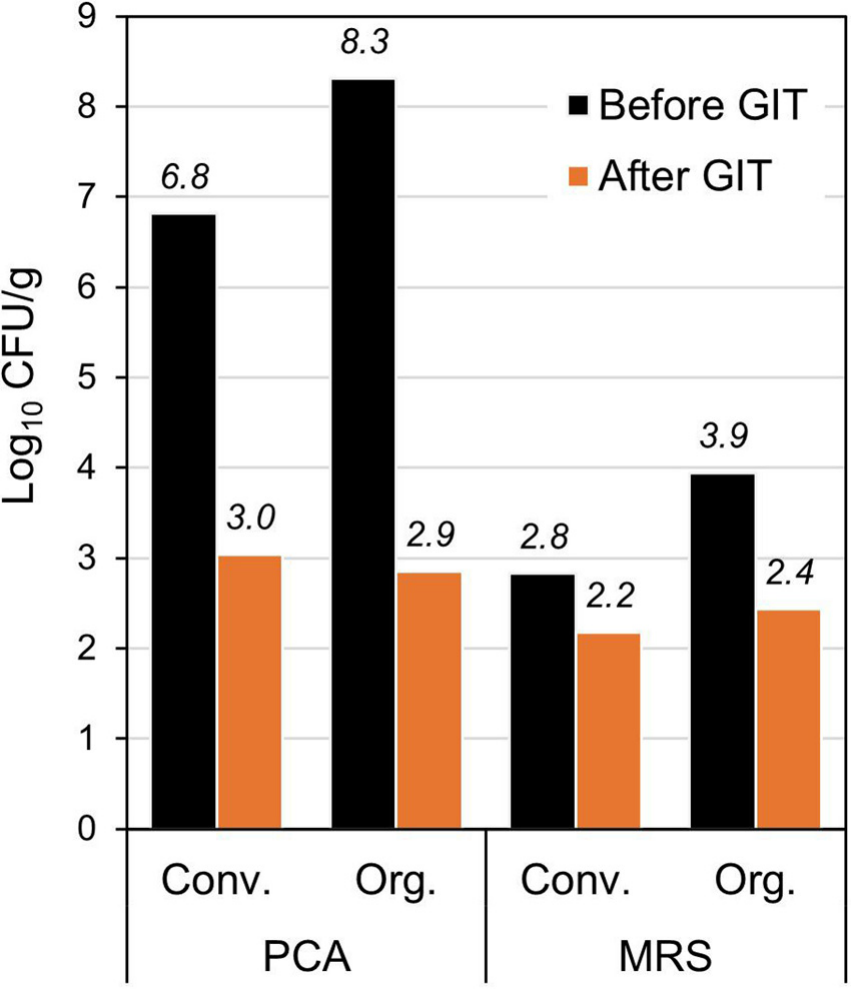
Viable counts in rocket salad before and after INFOGEST simulated gastrointestinal transit (GIT).

To assess the potential ability of bacteria colonizing rocket salads to survive passage through the gastrointestinal tract, we carried out viable bacterial counts of rocket samples before and after a static *in vitro* simulation of gastrointestinal digestion performed according to the INFOGEST protocol (18). Specifically, we used PCA and MRS agar as described in ISO protocols for the quantification of viable bacteria associated with two different commercial ready-to-eat rocket products produced by means of conventional and organic farming. Simulated gastrointestinal transit induced a drastic reduction in viable bacteria in both conventional and organic rocket salads according to the quantification on PCA (Fig. 8). On the contrary, the number of viable LAB quantified on MRS agar was much less reduced by simulated gastrointestinal digestion (Fig. 8).

Finally, we isolated several colonies from PCA plates inoculated with rocket samples treated with simulated gastrointestinal digestion. Next, taxonomic identification by 16S rRNA gene sequencing assigned the isolates to 8 different species: Kocuria arsenatis and Oerskovia enterophila (both of the order *Micrococcales*), Bacillus amyloliquefaciens and Priestia aryabhattai (both of the order *Bacillales*), and *Enterococcus* sp. in conventional rocket salads and Pseudomonas fragi, Pseudomonas sp., and *Rhodococcus* sp. in organic rocket salads.

Overall, the results of the experiments of simulated gastrointestinal digestion revealed that several taxonomically different bacteria associated with ready-to-eat rocket salad can survive gastrointestinal transit and that such an ability is particularly enhanced in rocket-associated LAB.

## DISCUSSION

This study was carried out under the hypothesis that the intake of raw unfermented food, especially vegetables, represents a nonnegligible source of live bacteria for the human gastrointestinal tract. To test this hypothesis, we selected rocket salad as a reference, mainly because it is cultivated worldwide and commercialized in many countries, where rocket leaves are most often eaten fresh and available in ready-to-use packages. Moreover, the consumption of rocket salad is suggested not only because it is a low-calorie vegetable and a good source of minerals and vitamins but also because it contains numerous bioactives, including glucosinolates, which are secondary plant metabolites that possess protective properties (19, 20). Here, we investigated whether this vegetable, consumed raw, could also be a relevant source of bacteria.

The metataxonomic analysis of several rocket salads from different producers purchased at local supermarkets in Milan, Italy, revealed the presence in all samples of a microbiota dominated by *Proteobacteria*, with Pseudomonas and *Flavobacterium* as the dominant genera. This result is in agreement with those of several other studies that found these bacteria to be the main members of the microbiota of different salads (21).

The overall bacterial community structures observed through β-diversity analysis were not significantly dissimilar between ready-to-eat and unwashed salads, letting us speculate that a significant part of the bacteria that we found may reside in rocket leaves as endophytes, as experimentally observed in other vegetables (21). On the contrary, profound diversity was found in the microbiota of traditionally, compared to vertically, farmed rocket salads in terms of both taxonomic composition and viable cell abundance. In addition, taxonomic diversity analysis revealed a significantly higher phylogenetic distance among the bacteria of vertical farming rocket samples (and among the samples collected from different trays from the same production site) than among the bacteria of traditional farming rocket samples. This result lets us speculate that in open-field farming, the host-level selection of environmental microbes shapes the rocket microbiome (as described previously for other plants [22]), whereas under the “clean” environmental conditions of vertical farming, far fewer microorganisms are available, leading to the formation of a rocket microbiome that is mostly a non-plant-specific representation of the environmental (soil) microbes.

Although their overall bacterial community structures (as assessed by α- and β-diversity analyses) were not significantly dissimilar, rocket samples from organic/integrated farming were distinguishable from conventional farming rocket salads by the differential abundances of several bacterial taxa. Interestingly, the 16S rRNA gene sequences found to be enriched in rocket samples from organic/integrated farming were principally ascribed to bacterial taxa known to be common members of the mammalian gastrointestinal microbiota, such as the families *Ruminococcaceae*, *Enterobacteriaceae*, and *Helicobacteraceae*. This result could potentially be explained by the reduced use of agricultural chemicals and the exploitation of manure for soil fertilization in organic farming systems compared to conventional farming systems, which rely more on the use of synthetic fertilizers, fungicides, and pesticides (23), potentially resulting in the long-term lowering of the taxonomic and phylogenetic richness, diversity, and heterogeneity of the soil microbiota (24, 25).

Notably, we observed that rocket salads from vertical farming carried levels of viable bacterial cells that were between 2 and 3 orders of magnitude lower than those of rocket salads from traditional farming. On the one side, this result supports the notion that vertical farming, also considering the highly controlled conditions of this agricultural system, could permit the production of vegetal products with potentially improved hygiene and safety profiles, in addition to the absence of undesired contaminants and residuals (26). Nonetheless, on the other side, the drastic reduction in viable microbial cells that occurs with vertical farming results mainly in the elimination of harmless bacteria that, according to the microbiome depletion hypothesis, might benefit health, constantly contributing to the shaping and maintenance of a eubiotic gastrointestinal microbiota across the host’s life span.

In this study, we focused our attention on lactic acid bacteria (LAB) associated with rocket salad because these microorganisms have been widely demonstrated to possess a wide range of peculiar abilities that can positively impact the gastrointestinal ecosystems of numerous animals (including humans), resulting in significant health benefits to the host at both the local and systemic levels (12). The phyllosphere of numerous vegetables is a well-known source of LAB (27, 28), which have been proposed as promising cultures to be exploited for food preservation and probiotic applications (29–32). In particular, more than 20% of all LAB isolates in our study belonged to Leuconostoc mesenteroides, a species that has been frequently shown to exert probiotic activities *in vitro* and *in vivo* (29, 33–36) and that includes several strains already employed in commercially available probiotic products. In addition, about 14% of LAB isolates were ascribed to *Weissella* species, a genus constituting bacteria frequently isolated from food that are emerging for their promising health-promoting activities (37), such as the demonstrated properties in the oral cavity for the treatment of halitosis (38). We also isolated several other strains belonging to LAB species with well-recognized health-promoting (probiotic) capabilities, including the ability to survive gastrointestinal transit, such as Lactiplantibacillus plantarum (39), Lactococcus lactis (40), Latilactobacillus sakei (41), and Levilactobacillus brevis (42). Notably, the experiment of simulated orogastrointestinal digestion revealed the distinctly enhanced ability of rocket-associated LAB to survive gastrointestinal transit compared to the other bacterial members of the microbiota. This result supports the hypothesis that the LAB ingested with rocket salad can reach the human gut alive, where they can potentially contribute to microbiome activities and influence host health.

Besides LAB, simulated digestion experiments suggested that rocket salads may also vehiculate into the human gastrointestinal tract viable bacterial cells belonging to numerous other different taxa, including endophytes (e.g., Kocuria arsenatis) and common soil inhabitants (e.g., Bacillus amyloliquefaciens), both Gram-negative and spore-forming bacteria. Reportedly, these environmental bacteria possess immunostimulatory properties that, according to the microbiome depletion hypothesis, can contribute to the establishment of immune tolerance and the maintenance of immune homeostasis (1, 43–45). In this study, the bacteria in rocket microbiota were assessed using a DNA-based methodology, and therefore, live or dead bacteria were not distinguished. Furthermore, as also suggested by simulated digestion experiments, most bacteria do not survive gastrointestinal transit. However, reportedly, the immunomodulatory/immunostimulatory activities of microbial cells may take place independently from cell viability (46), prevalently in the ileum (47), through the stimulation of host pattern recognition receptors (PRRs) by means of microbe-associated molecular patterns (MAMPs) such as lipopolysaccharides, murein, and lipoteichoic acids, etc. (48).

### Conclusion.

Accumulating experimental data suggest that modern Western-type diets, which are composed of foods with reduced amounts of naturally occurring microorganisms, may permanently damage health by also inducing the loss of key microbial taxa within the gastrointestinal microbiota (2, 49). Considering the results obtained in this study, it may be speculated that the intake of fresh rocket salad from ready-to-eat commercial products may be a source of live bacteria that possess the ability to survive gastrointestinal transit, potentially contributing to counteracting microbial depletion occurring in industrialized societies. We believe that the deductions resulting from this study’s findings can be valid for virtually all plant foods that are consumed raw. Human dietary intervention studies are needed to confirm the hypothesis that raw foods, especially vegetables, can contribute significantly to the shaping and maintenance of the microbial community structure of human orogastrointestinal ecosystems.

## MATERIALS AND METHODS

### Samples.

**FIG 9:**
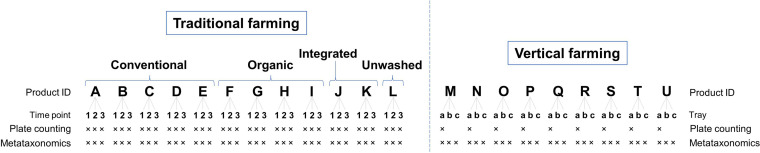
Study scheme and summary of the rocket samples investigated. See the text for details.

A representation of the analyzed rocket salad samples, farming methods (vertical, conventional, organic [Org], integrated [Int], and conventional/nonwashed [CNW]), and the study scheme are reported in Fig. 9. To summarize, 12 commercial rocket salad products (indicated with the letters A to L in Fig. 9) were purchased three times each from different supermarkets in Milan at 1-week intervals (1, 2, and 3 in Fig. 9), whereas 9 different cultivars of rocket salad produced through vertical farming methods (indicated with the letters M to U in Fig. 9) were collected directly from the producer (Agricola Moderna, Melzo, Italy) in triplicate (i.e., leaves collected from three different trays per cultivar at the production site) (a, b, and c in Fig. 9) in the same period.

### DNA extraction from rocket salads and 16S rRNA gene profiling.

Ten grams of rocket leaves was frozen in liquid nitrogen, ground with a mortar and pestle, and immediately processed or stored at −80°C. Starting from 250 mg of ground rocket salad, total (microbial and plant) DNA was extracted using the PowerLyser PowerSoil kit (Qiagen, Milan, Italy) according to the manufacturer’s instructions. The quality of the extracted DNA was evaluated by the absorbance at 260/280 nm and 260/230 nm. The DNA was quantified by fluorescence analysis using the Qubit broad-range kit (Thermo Fisher Scientific, Waltham, MA, USA). The V3 and V4 regions of the 16S rRNA gene were sequenced using the Illumina MiSeq platform with plant-blocking oligonucleotides (50) (IGA Technology Services, Udine, Italy). Sequencing reads were analyzed by means of the Quantitative Insights into Microbial Ecology 2 (QIIME 2) version 2021.8 bioinformatic pipeline with the Divisive Amplicon Denoising Algorithm (DADA2) (51) using the Greengenes database (version 13_5) for taxonomic assignments to amplicon sequence variants (ASVs).

### Total mesophilic and lactic acid bacterium counts.

The counts of mesophilic bacteria and lactic acid bacteria (LAB) were determined according to the ISO 4833 (2013) and ISO 15214 standard protocols. Briefly, 10-g samples were homogenized in 90 g of saline peptone water (SPW) (1.0 g/L peptone, 8.5 g/L NaCl [pH 7.0]) in a Stomacher 3500 peristaltic homogenizer (Seward, West Sussex, United Kingdom) for 2 min. The samples were then diluted through decimal serial dilutions, taking 1 mL of the suspension and diluting it in 9 mL of SPW. Subsequently, 1 mL of each suspension was plated by pour plating onto plate count agar (PCA) for the total counts (Sharlab, Riozzo di Cerro al Lambro, Milan, Italy) or de Man-Rogosa-Sharpe (MRS) agar (Difco, Detroit, MI, USA) at pH 5.7. Finally, the plates were incubated at 30°C for 72 h under aerobic conditions.

### Isolation and taxonomic characterization of lactic acid bacteria.

Single colonies grown on MRS plates were isolated with a loop and streaked onto MRS agar. This step was repeated twice, and single colonies were then inoculated into MRS broth. Both agar plates and broth were inoculated at 30°C for 48 h. One milliliter of the bacterial cell suspension was centrifuged at 14,000 × *g* for 5 min. The pellet was washed twice with sterile phosphate-buffered saline (PBS) (1.0 g/L NaCl, 0.2 g/L KCl, 1.44 g/L Na_2_HPO_4_, 0.24 g/L KH_2_PO_4_ [pH 7.4]) and resuspended in 500 μL of sterile PBS. The cell suspension was then heated at 99°C for 4 min, ice shocked for 2 min, and heated again at 99°C for 2 min. The cell lysate was used directly for PCR using the universal primers P0 (5′-GAGAGTTTGATCCTGGCTCAG-3′) and P6 (5′-CTACGGCTACCTTGTTACGA-3′) (52) to amplify the 16S rRNA gene. Amplicons were then sequenced using primer 1100R (5′-GGGTTNCGNTCGTTG-3′) (53). Taxonomic assignment was carried out by a BLASTN search of the GenBank rRNA database. A species designation was assigned to isolates only for similarities of >98%.

### Simulated gastrointestinal digestion.

The INFOGEST protocol for static *in vitro* simulation of gastrointestinal food digestion (18) was used to evaluate the survival of rocket salad-associated microbes during transit through the orogastrointestinal tract. Briefly, 5 g of rocket salad was homogenized with 4 g of simulated salivary fluid [15.1 mM KCl, 3.7 mM KH_2_PO_4_, 13.6 mM NaHCO_3_, 0.15 mM MgCl_2_(H_2_O)_6_, 0.06 mM (NH_4_)_2_CO_3_, 1.1 mM HCl, 1.5 mM CaCl_2_(H_2_O)_2_] in a Stomacher 3500 peristaltic homogenizer. Next, α-amylase from Aspergillus oryzae (Merck, Milan, Italy) was added to a final concentration of 75 U/mL. The suspension was diluted to 10 mL with deionized water and then incubated at 37°C for 2 min under continuous shaking. Eight milliliters of simulated gastric fluid [6.9 mM KCl, 0.9 mM KH_2_PO_4_, 25 mM NaHCO_3_, 47.2 mM NaCl, 0.12 mM MgCl_2_(H_2_O)_6_, 0.5 mM (NH_4_)_2_CO_3_, 15.6 mM HCl, 0.15 mM CaCl_2_(H_2_O)_2_] was then added, and the pH was corrected to 3.0 using 5 M HCl. Porcine pepsin (Merck) was added to a final concentration of 2,000 U/mL, and rabbit gastric extract (RGE; Lipolytech, Marseille, France) was added to a final concentration of 60 U/mL of lipase. After dilution to 20 mL with deionized water, samples were incubated at 37°C for 2 h under continuous shaking. Eight milliliters of simulated intestinal fluid [6.8 mM KCl, 0.8 mM KH_2_PO_4_, 85 mM NaHCO_3_, 38.4 mM NaCl, 0.33 mM MgCl_2_(H_2_O)_6_, 8.4 mM HCl, 0.6 mM CaCl_2_(H_2_O)_2_] was added, and the pH was corrected to 7.0 using 5 M NaOH. Porcine pancreatin (Merck) and bovine bile salts (Merck) were added to final concentrations of 100 U/mL of trypsin in pancreatin and 10 mM bile salts. Subsequently, the volume was adjusted to 40 mL, and the samples were incubated at 37°C under shaking for 2 h. The volume was then adjusted to 50 mL with SPW, and the samples were diluted, plated, and incubated as described above on both PCA and MRS agar plates. Colonies on PCA plates were isolated and taxonomically identified by sequencing of 16S rRNA gene amplicons as described above.

### Statistical analysis.

Statistical computing was carried out using the R programming language (version 3.4.2) and GraphPad Prism version 8. An unpaired *t* test was used to compare the viable bacterial counts (expressed as CFU per gram) of the different sample sets using a false discovery rate (FDR) of 1% without assuming consistent standard deviations. The number of observed taxonomic features, the Shannon H index, and Pielou’s evenness were calculated to assess the α-diversity. UniFrac algorithms were used to study the intersample diversity of the fecal microbiota composition. Differences in bacterial taxonomic compositions between groups were defined using the LDA effect size (LEfSe) algorithm considering an α value of 0.5 and a log_10_ LDA score of >2 (54).

### Data availability.

The FASTQ data generated during 16S rRNA gene profiling have been deposited in the European Nucleotide Archive (ENA) of the European Bioinformatics Institute under accession number PRJEB54675. All data supporting the findings of this study are openly available in Dataverse at https://dataverse.unimi.it/dataverse/VegMicroEcol.
