# Supplementary material for: Ready-To-Eat Rocket Salads as Potential Reservoir of Bacteria for the Human Microbiome
Source: Microbiol Spectr. 2022 Dec 20;11(1):e02970-22. doi: 10.1128/spectrum.02970-22 (PMC9927460; doi:10.1128/spectrum.02970-22)

Ready-to-eat rocket salads as potential reservoir of bacteria for the human microbiome

Giacomo Mantegazza[1], Giorgio Gargari[1], Robin Duncan[1], Fabio Consalez[1], Valentina Taverniti[1], Patrizia Riso[2], Simone Guglielmetti[1]*

[1] Division of Food Microbiology and Bioprocesses and [2] Division of Human Nutrition, Department of Food, Environment, and Nutritional Science, Università degli Studi di Milano, Milan, Italy

*Correspondence: simone.guglielmetti@unimi.it

Supplementary Fig. S1. Genus-level composition of the bacterial community associated to rocket salad leaves. Sample labels are according to the scheme represented in Fig. 1.

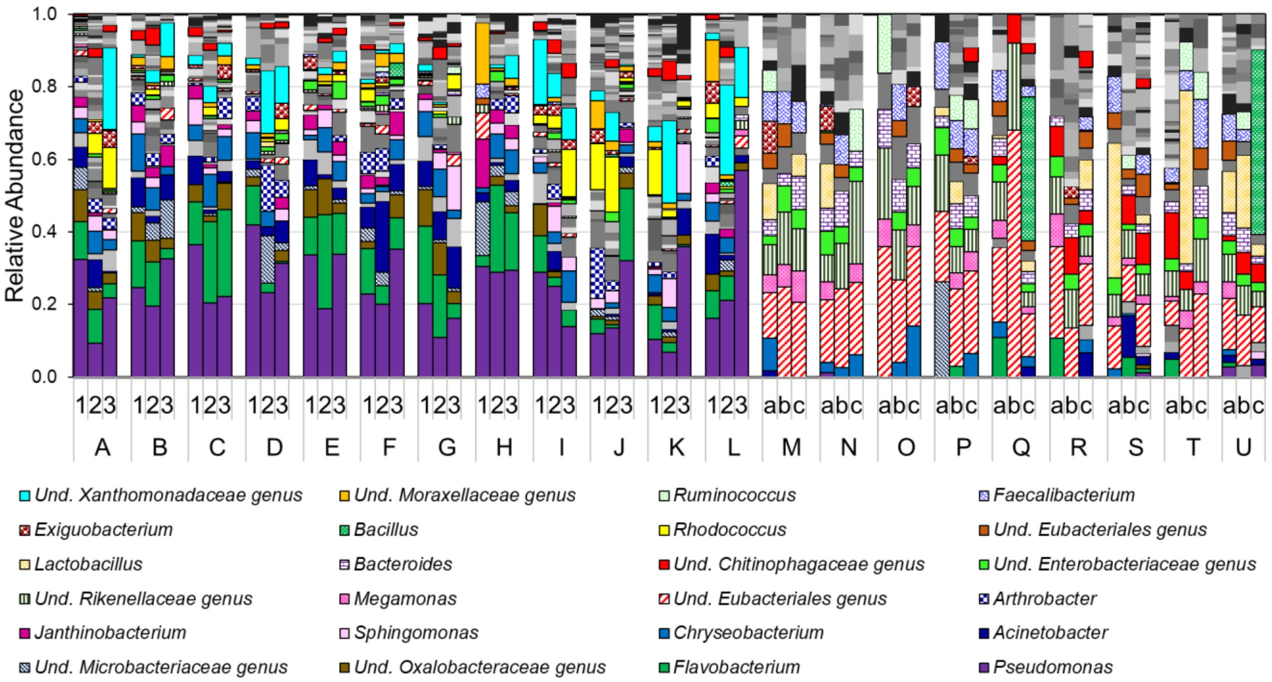

Supplementary Fig. S2. Summary of the species of lactic acid bacteria isolated from rocket salads. The precise number of bacterial isolates is indicated Sample labels are according to the scheme represented in Fig. 1. Background colors constitute a heatmap that represents the number of isolates.

	Conventional															Organic												Integrated						UW		
	A			B			C			D			E			F			G			H			I			J			K			L		
**Species**	1	2	3	1	2	3	1	2	3	1	2	3	1	2	3	1	2	3	1	2	3	1	2	3	1	2	3	1	2	3	1	2	3	1	2	3
Lactiplantibacillus plantarum													1																					1		
Lactococcus lactis											1									5			6									1				
Latilactobacillus graminis													3																							
Latilactobacillus sakei						1	3	1			1		1							2											2			8	3	
Leuconostoc carnosum																		1																		
Leuconostoc citreum															8																					
Leuconostoc gelidum					2			1				7	4	2				1		2			4		4		2								1	8
Leuconostoc holzapfelii															4																					
Leuconostoc mesenteroides			2	1	2			3	15		2	1		4	1	1	2		2	4			3						2					4	1	4
Leuconostoc miyukkimchii					2			1				1	3	6				2	4				1		3	2	4				1			1	1	1
Leuconostoc rapi																											6									
Levilactobacillus brevis																10			2												6					
Paucilactobacillus nenjiangensis															1																					
Paucilactobacillus oligofermentans															1																					
Weissella cibaria																			2																2	
Weissella koreensis																																			4	
Weissella oryzae																																4				
Weissella soli					2							5		2				1		2			1				3		2						2	2
Herbaspirillum huttiense																										2										
**Total isolates per sample**		**2**			**10**			**24**			**18**			**41**			**18**			**25**			**15**			**26**			**4**			**14**			**43**	
**Total isolates per farming type**	**95**															**84**												**18**						**43**		

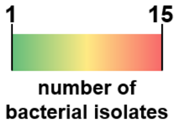

Supplement: Supplemental file 1 — Figures S1 and S2. Download spectrum.02970-22-s0001.pdf, PDF file, 0.4 MB [file spectrum.02970-22-s0001.pdf]
